# Very long-chain saturated fatty acids in plasma lipids: association with cardiometabolic risk influenced by lipid interactions

**DOI:** 10.1186/s12933-025-03037-4

**Published:** 2025-12-22

**Authors:** Inés Domínguez-López, Fabian Eichelmann, Marcela Prada, Matthias B. Schulze

**Affiliations:** 1 Department of Molecular Epidemiology, German Institute of Human Nutrition Potsdam-Rehbruecke, Nuthetal, Germany; 2 German Center for Diabetes Research (DZD), Munich-Neuherberg, Germany; 3 Institute of Nutritional Science, University of Potsdam, Nuthetal, Germany

**Keywords:** Lipidomics, Cardiometabolic, Lipid network, Type 2 diabetes, Cardiovascular disease, Fatty acids

## Abstract

**Background:**

Very long-chain saturated fatty acids (VLCSFA) may influence cardiometabolic health differently from other, often detrimental, saturated fatty acids (SFA). Evidence remains inconclusive, partly because VLCSFA are metabolically derived from SFA, making it difficult to disentangle their individual effects due to potential confounding of correlated lipids. Prior studies rarely accounted for correlations with other lipids or do not consider VLCSFA-specific lipid classes. We investigated prospective associations of circulating VLCSFA (C20:0, C22:0, C24:0) across multiple plasma lipid classes with type 2 diabetes (T2D) and cardiovascular disease (CVD), accounting for confounding by correlated lipids.

**Methods:**

We constructed two nested case-cohort studies within the European Prospective Investigation into Cancer and Nutrition (EPIC)-Potsdam cohort: 1911 in the T2D case-cohort (774 cases); 1704 in the CVD case-cohort (547 cases). Plasma concentrations of VLCSFA were measured in 12 lipid classes. A data-driven network including SFA across all lipid classes was used to identify precursors and downstream lipid metabolites for each lipid class of VLCSFA. The correlated lipids were gradually incorporated in multivariable-adjusted Cox regression models between individual lipids and disease risk.

**Results:**

C20:0 was distributed across more lipid classes than C22:0 and C24:0. After including all correlated precursors and downstream lipid metabolites in the model, we observed that higher C22:0 levels were linked to higher T2D risk, while associations for C20:0 and C24:0 varied by class. Ceramides C20:0 (hazard ratio [HR] per SD: 0.52, 95% CI 0.35–0.79) and C24:0 (0.46, 0.27–0.79) were inversely associated with T2D, whereas dihydroceramides C20:0 (1.36, 1.07–1.72) and sphingomyelin C24:0 (1.61, 1.15–2.26) showed positive associations. Monoglycerides and cholesteryl esters containing VLCSFA were associated to higher risk of both outcomes. Most of these relationships were not observed when the confounding or mediation by correlated lipids was not considered.

**Conclusions:**

VLCSFA show different metabolic roles in cardiometabolic diseases and highlight the importance of adjusting for confounding by correlated lipids. These findings challenge the traditional view that SFA exert uniform negative effects and suggest class-specific VLCSFA profiles may improve risk prediction of cardiometabolic diseases, guiding more precise prevention strategies.

**Supplementary Information:**

The online version contains supplementary material available at 10.1186/s12933-025-03037-4.

## Research insights

**What is currently known about this topic?**

Saturated fatty acids are generally considered harmful to cardiometabolic health. However, the role of very-long-chain saturated fatty acids (VLCSFA) remains unclear, as they may exert different biological effects compared to their saturated fatty acids precursors.

**What is the key research question?**

How are very-long-chain saturated fatty acids (VLCSFAs) from different lipid classes associated with type 2 diabetes and cardiovascular disease when their relationships are disentangled from the confounding and mediating effects of correlated lipids?

**What is new?**

We developed a data-driven network to identify lipids correlated with each VLCSFA across all lipid classes and incorporated these correlated lipids stepwise into adjustment models. Associations between VLCSFAs and type 2 diabetes or cardiovascular disease became apparent only after controlling for correlated precursor and downstream lipids. This approach revealed that these relationships differ by fatty acid chain length and lipid class.

**How might this study influence clinical practice?**

This study highlights that VLCSFA associations with disease depend on the specific lipid classes carrying them, cautioning against treating saturated fats as a single uniform group and suggesting that class-specific VLCSFA measures could enhance risk assessment and prevention strategies for type 2 diabetes and cardiovascular disease.

## Introduction

Saturated fatty acids (SFA) have traditionally been associated with increased risk of cardiovascular disease (CVD), leading to recommendations to limit their intake [1]. However, emerging evidence shows that not all SFA affect cardiometabolic health equally, as their effects may vary depending on the chemical structure. Very long-chain saturated fatty acids (VLCSFA)—including arachidic acid (C20:0), behenic acid (C22:0), and lignoceric acid (C24:0)—have gained interest due to their potentially different effects on health [2]. Although VLCSFA are found in foods such as peanuts and certain plant oils [3], their dietary intake is generally low. Consequently, most VLCSFA are produced endogenously through the elongation of their SFA precursors like palmitic acid (C16:0) and stearic acid (C18:0) within the endoplasmic reticulum [4]. Because SFA like C16:0 are known to have harmful effects on health [5], they can confound the relationships between VLCSFA and disease risk.

VLCSFA are mainly found in sphingolipids such as ceramides and sphingomyelins [2]. Studies examining plasma phospholipids and red blood cell membranes suggest that VLCSFA may be inversely associated with type 2 diabetes (T2D) risk across different cohorts [6, 7]. Data pooled from 12 cohorts by the Fatty Acids and Outcomes Research Consortium (FORCE) showed this protective association [8]. However, they only adjusted for C16:0, overlooking the potential confounding effects from other fatty acids. Regarding CVD, plasma phospholipid VLCSFA have been linked to lower risks of heart failure, atrial fibrillation, and coronary heart disease in diverse populations, with studies also adjusting for C16:0 [9, 10]. VLCSFA are produced by elongation of SFA, and their tightly linked circulating levels may introduce confounding that influences these relationships. However previous studies have not systematically considered intercorrelation patterns among VLCSFA and with other SFA and may not have sufficiently controlled confounding by correlated fatty acids.

Furthermore, because analyses typically focus on total plasma phospholipids or red blood cell membranes, they overlook structural and functional differences among phospholipid subclasses that can lead to different effects on disease risk. To date, VLSCFA research has focused on circulating ceramides given their established links to cardiometabolic risk, but results have been inconsistent [11–13]. Prior studies generally did not account for correlation among lipid species; when addressed, it was often limited to ratios (normalization to 16:0) [11, 12] and rarely considered VLCSFA‑containing species across multiple lipid classes [13]. Moreover, other plasma lipid classes containing VLCSFA, e.g. glycerolipids and cholesterol esters have been understudied.

This study investigates the associations between plasma concentrations of VLCSFA across different plasma lipid classes and cardiometabolic outcomes while accounting for the influence of other SFA. By constructing a data-driven network based on conditional dependencies among SFA, our approach aims to disentangle the specific roles of individual VLCSFA from those of correlated lipid species, thereby clarifying their unique contributions to cardiometabolic risk.

## Methods

### Study design

The European Prospective Investigation into Cancer and Nutrition (EPIC) Potsdam cohort study enrolled 27,548 men and women from the general population around Potsdam, Germany, between 1994 and 1998. At baseline, the study collected blood samples, information on prevalent diseases, sociodemographic characteristics, and lifestyle factors, and anthropometric and blood pressure measurements following a standardized protocol [14, 15]. Blood plasma samples were stored either in liquid nitrogen tanks at – 196 °C or in deep freezers at – 80 °C until analysis. Participants were actively followed up every 2 to 3 years using follow-up questionnaires to identify incident cases of chronic diseases, with response rates exceeding 90%. Consent was obtained from all participants, and approval was given by the ethics committee of the State of Brandenburg, Germany [16].

Cases of incident T2D and CVD were self-report of a respective diagnosis, death certificates, tumor centers, and clinical records linkage. For T2D, pharmacological treatment or dietary changes reported during follow-up were also considered. Additionally, death certificates and data from tumor centers, physicians, or clinics providing assessments for other conditions were reviewed for signs of new T2D cases. Verification of potential cases was carried out through standardized forms filled by the participants’ treating physicians. Only physician-verified cases of T2D (ICD-10 code E11), MI (ICD-10 code I21) and stroke (ischemic: ICD-10 I63.0–I63.9, hemorrhagic: ICD-10 I60.0–I61.9 and undetermined stroke: ICD-10 I64.0–I64.9]) with a diagnosis date after the baseline examination were considered confirmed incident cases. Participants who experienced silent cardiovascular events, which were not documented within 28 days after they occurred, were excluded from all analyses due to nonverifiable data.

The present study used a case-cohort design, incorporating all identified cases of CVD and T2D and a randomly selected subcohort for efficient study of molecular phenotypes [17]. The random subsample (subcohort, n = 1,262) was chosen from the baseline participants who provided blood (n = 26,437), which served as a common reference population for both end points. For each end point, all incident cases occurring in the full cohort by a specified censoring date (November 30, 2006, for CVD, and August 31, 2005, for T2D) were included in the analysis. After excluding prevalent cases of T2D, the analytic sample for T2D involved 1,911 participants, including 774 incident cases (26 part of the subcohort). After excluding prevalent cases of CVD, a total of 1,704 participants including 547 incident cases (29 part of the subcohort), were included in the CVD analyses. Follow-up time in the study was defined as the period between enrollment and the point of study exit, which was determined by the diagnosis of the respective disease, death, dropout, or the final censoring date, whichever occurred first. A flow-chart of the study design can be found in Additional file 1: Fig S1.

### Lipidomics analyses

Lipidomics in plasma samples collected at baseline were performed by Metabolon using the Metabolon Complex Lipid Panel as previously described [17]. Further details are described in Additional file 1: Expanded Methods.

The Complex Lipid Panel generated measurements for 15 lipid classes: free fatty acids (FFA), cholesteryl esters (CE), monoacylglycerols (MG), ceramides (Cer), dihydroceramides (dhCer), lactosylceramides (LacCer), hexosylceramides (HexCer), sphingomyelins (SM), lysophosphatidylethanolamines (LPE), lysophosphatidylcholines (LPC), diacylglycerols (DG), triacylglycerols (TG), phosphatidylcholines (PC), phosphatidylethanolamines (PE)—including phosphatidylethanolamine ethers (PEOs) and phosphatidylethanolamine plasmalogens (PEPs)—and phosphatidylinositol (PI). VLCSFA were not detected in LPE, LPC, or PI. For the present analysis, a total of 57 fatty acid sums within class, ranging from C16:0-C26:0, were used. Within-class FA sum refers to summing all concentrations of molecular species containing a specific FA within a lipid class. Within-class FA sums are synonymous with molecular species level in lipid classes containing only one variable FA per molecule (FFA, CE, MG, Cer, dhCer, LacCer, HexCer, SM, LPE, LPC).

Lipid class sums were calculated by summing the concentrations of all molecular species belonging to the same class. Total fatty acid sums were computed by summing the concentrations of all molecular species that contained a specific fatty acid, regardless of lipid class.

### Statistical analyses

Plasma lipids with > 70% of missing values were excluded. Among the remaining ones, those with missing values corresponding to concentrations below the limit of quantification were imputed using the quantile regression imputation of left-censored data approach from the R package imputeLCMD. All lipidomics variables were log-transformed and z-scaled (mean = 0, SD = 1) to allow comparison of association strength across lipids and to stabilize skewed distributions.

We constructed a data-driven network of conditional dependencies to explore interrelationships among VLCSFA, their metabolic precursors, and downstream metabolites based on lipidomic profiles. We included lipids with C16:0 and C18:0 to account for precursors generated by de novo lipogenesis, as well as C26:0 to capture immediate elongation products (e.g., of C24:0). Edges indicate lipid pairs that remain dependently correlated after conditioning on subsets of the other lipids. We estimated the network using an order‑independent implementation of the PC algorithm provided in the NetCoupler R package, which iteratively removes an edge when some conditioning set renders the pair conditionally independent [13]. The resulting graph is the undirected skeleton of a causal directed acyclic graph consistent with the observed conditional independence structure and does not assign directions. For each lipid, we defined direct neighbors as those connected by edges, distinguishing three groups: (1) precursor neighbors within the same class with shorter chain lengths; (2) all neighbors within the same class; and (3) all neighbors including those from other classes. These neighbor groups were progressively included as covariates in the adjustment models to account for the lipid intercorrelation and estimate their direct effects not confounded or mediated by up- or downstream lipids.

Cox proportional hazard models with Prentice weights (to account for oversampling cases in the case-cohort design) [18] were used to calculate the hazard ratios (HR) and their 95% confidence interval (CI) for the risk of T2D and CVD outcomes associated with the plasma lipid concentrations as continuous variables (per 1-SD). Age was the underlying time variable, with entry time as age at baseline and exit time as age at event or censoring. Four models were applied to gradually adjust the associations for the correlated lipids. The *basic* adjustment model included age (continuous), sex (men, women), waist circumference (continuous), height (continuous), leisure-time physical activity (continuous), smoking status (never [no reported history of smoking], former [not currently smoking but with a prior history], and current smoker [smoking at baseline]), alcohol intake (none, low [0.1–6 g/d], moderately low [6.1–12.0 g/d], moderately high [12.1–24.0 g/d], high [24.1–60.0 g/d], very high [60.1–96.0 g/d], and extremely high [> 96.1 g/d]), highest achieved education level (primary school, secondary school/high school and college/higher education degree), fasting status at blood draw (overnight fast, only drink and unfasted), total energy intake (continuous), plasma total cholesterol and TG (continuous), and antihypertensive, lipid-lowering, and acetylsalicylic acid medication (yes, no). Models for incident CVD were additionally adjusted for prevalent T2D (yes, no) and the proportion of glycohemoglobin (continuous) to capture clinical disease status (which may be controlled by treatment or lifestyle) and glycemic control to reduce residual confounding from undiagnosed (pre)diabetes. The *only-precursors* model was further adjusted for precursor lipids—defined as those directly connected in the data-driven network with shorter chain length—within the same class to assess confounding by their precursors. The *all-class* model included all direct neighbors from the same lipid class, thereby accounting for downstream metabolites that could potentially act as mediators to better estimate the direct effect of each lipid. The *all-neighbors* model was further adjusted for all direct neighbors across all lipid classes, accounting for confounders and mediators throughout the entire lipid network. TG and CE were not adjusted for clinical measures of TG and CE, respectively. All analyses were adjusted for the respective class sum to separate the association of the VLCSFA-containing lipids from the overall association of its lipid class sum. To ensure consistency in the analysis, when lipids from different classes were included in the *all-neighbors* model, its corresponding lipid class sum was also incorporated. We evaluated effect modification by sex by including lipid-by-sex interaction terms in the adjusted models. For lipids with statistically significant interactions, we presented sex-stratified estimates. We tested for nonlinearity using restricted cubic splines and likelihood‑ratio tests comparing spline to linear models. We found no significant deviation from linearity; the spline curves were essentially flat across the exposure range. Accordingly, we report linear (per‑SD) associations.

Corrections for multiple testing were addressed by controlling the false discovery rate (FDR) separately for each outcome [19]. All analyses were performed with R (version 4.1.0).

## Results

### Participants characteristics

Baseline characteristics of participants in the EPIC-Potsdam case-cohort studies for T2D and CVD are presented in Table 1. On average, participants with incident T2D and CVD were older, more likely to be men, had larger waist circumference, higher prevalence of smoking, higher total energy intake, and more frequent use of medications compared to the subcohort.

**Table 1:** Baseline characteristics of the EPIC-Potsdam participants included in the T2D and CVD case-cohort

Characteristic	Subcohort CVD	Incident CVD Cases	Subcohort T2D	Incident T2D Cases
	N = 1,157^*1*^	N = 547^*1*^	N = 1,137^*1*^	N = 774^*1*^
Sex				
Men	444 (38%)	360 (66%)	448 (39%)	449 (58%)
Women	713 (62%)	187 (34%)	689 (61%)	325 (42%)
Age, years	49.6 (42.2, 57.8)	57.9 (52.3, 62.3)	49.4 (42.1, 57.6)	56.5 (49.5, 61.0)
Waist circumference, cm	85.0 (75.0, 94.0)	93.0 (85.0, 101.0)	85.0 (75.0, 93.5)	100.0 (92.0, 107.5)
Prevalent T2D	47 (4.1%)	4 (0.7%)		
Physical activity, METS/day	4.5 (2.0, 8.0)	5.0 (2.0, 10.0)	5.0 (2.0, 8.0)	4.5 (1.5, 8.5)
Educational level				
Primary school	443 (38%)	223 (41%)	439 (39%)	354 (46%)
Secondary/High school	276 (24%)	134 (24%)	271 (24%)	182 (23%)
College/Higher	438 (38%)	190 (35%)	427 (37%)	238 (31%)
Smoking habit				
Never	558 (48%)	174 (32%)	552 (49%)	266 (35%)
Former	364 (32%)	181 (33%)	358 (31%)	343 (44%)
Current	235 (20%)	192 (35%)	227 (20%)	165 (21%)
Total energy intake, KJ/day	8,416.1 (6,796.0, 10,240.4)	8,870.6 (7,276.0, 10,519.8)	8,446.4 (6,790.5, 10,300.2)	8,813.0 (7,164.5, 10,722.8)
Antihypertensive medication	222 (19%)	190 (35%)	227 (20%)	304 (39%)
Lipid-lowering medication	52 (4.5%)	37 (6.8%)	58 (5.1%)	81 (10%)
Acetylsalicylic acid medication	101 (8.7%)	53 (9.7%)	114 (10%)	100 (13%)
Alcohol intake				
None	31 (2.7%)	33 (6.0%)	32 (2.8%)	28 (3.6%)
Low	460 (40%)	188 (34%)	447 (39%)	288 (37%)
Moderately low	223 (19%)	97 (18%)	221 (19%)	156 (20%)
Moderately high	224 (19%)	98 (18%)	221 (19%)	138 (18%)
High	193 (17%)	108 (20%)	191 (17%)	134 (17%)
Very high	26 (2.2%)	23 (4.2%)	25 (2.2%)	30 (3.9%)
Systolic pressure, mmHg	128.0 (117.0, 139.5)	137.5 (126.5, 151.5)	128.0 (117.0, 140.0)	138.0 (127.7, 150.5)
Diastolic pressure, mmHg	83.0 (76.5, 90.0)	87.5 (81.5, 95.0)	83.0 (76.5, 90.0)	89.0 (82.5, 95.5)
Total cholesterol, mg/dL	205.4 (178.1, 230.1)	213.9 (190.7, 238.5)	204.6 (177.9, 230.2)	212.5 (186.6, 239.5)
Triglycerides, mg/dL	107.0 (75.6, 164.3)	143.0 (96.3, 204.3)	106.8 (75.1, 163.2)	170.2 (127.9, 240.6)
Hemoglobin A1c, %	5.4 (5.1, 5.8)	5.7 (5.4, 6.1)	5.4 (5.1, 5.7)	6.1 (5.7, 6.7)

^1^Values are shown as n (%) or median (Q1; Q3)

CVD, cardiovascular disease; T2D, type-2 diabetes; METS, metabolic equivalent of task

### Distribution of VLCSFA in lipid classes and intercorrelation patterns

**Fig. 1:**
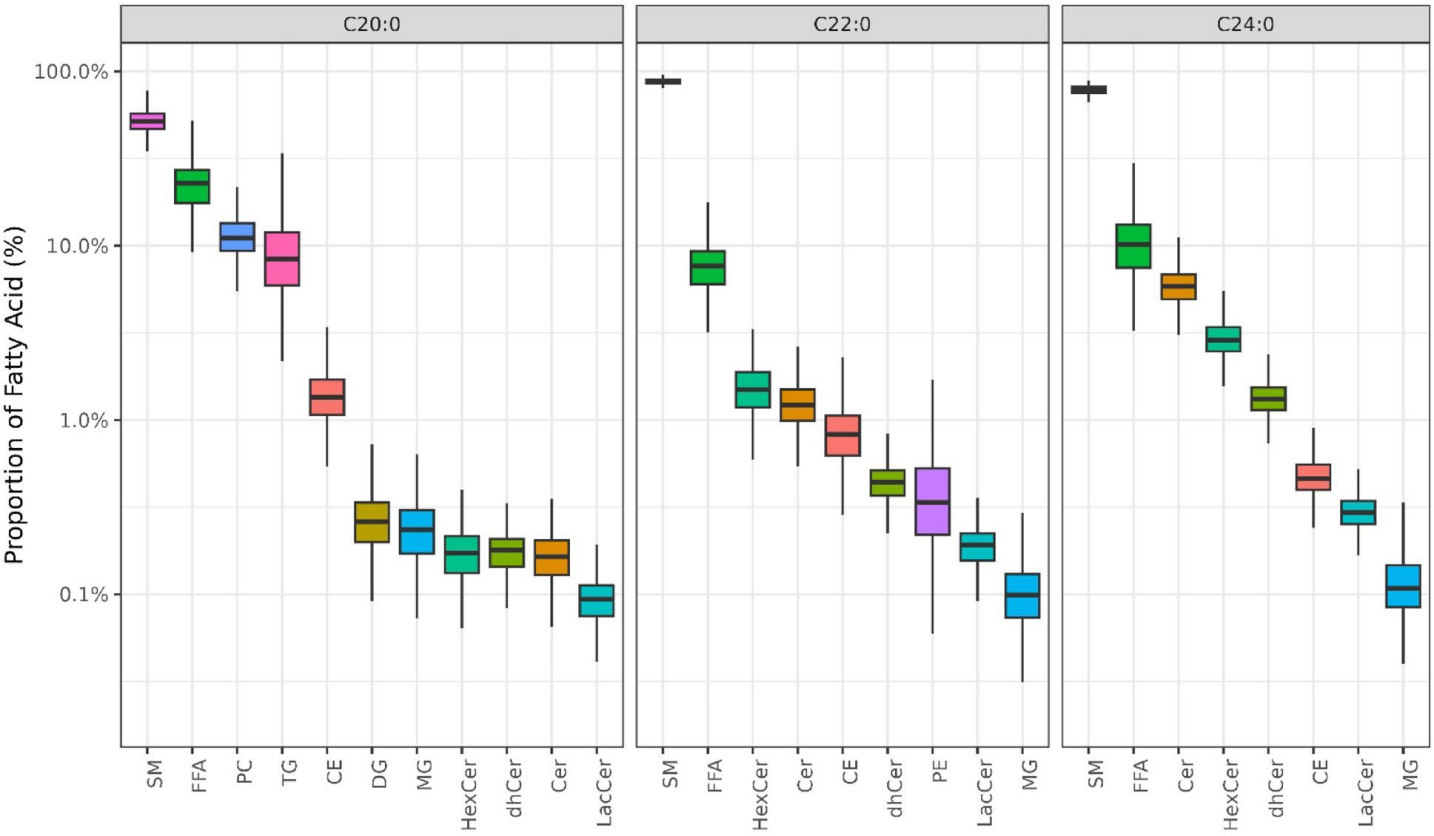
Distribution of lipid-classes across individual VLCSFA. CE indicates cholesteryl esters; Cer, ceramides; DG, diacylglycerols; dhCer, dihydroceramides; FFA, free fatty acids; HexCer, hexosylceramides; LacCer, lactosylceramides; MG, monoacylglycerols; PC, phosphatidylcholines; PE, phosphatidylethanolamines; SM, sphingomyelins; TG, triacylglycerides; and VLCSFA, very long chain saturated fatty acids

Figure 1 shows the distribution of VLCSFA across lipid classes, representing the proportion of each lipid class within the total amount of each respective fatty acid. SM was the predominant lipid class containing these VLCSFA, followed by FFA. For C20:0, LacCer was the least abundant lipid class, whereas for C22:0 and C24:0, the lowest proportions were observed in MG. C24:0 was relatively abundant in Cer and HexCer, and absent from TG, PE, PC, and DG. C20:0 was abundant in PC compared to other lipid classes, a pattern not observed for C22:0 and C24:0. Additionally, C22:0 was present in PE, unlike the other two VLCSFA.

For C20:0, the strongest correlations between lipid classes were observed between FFA and MG (r = 0.60), and between DG and TG (r = 0.48, Additional file 1: Fig. S2). For C22:0 and C24:0, although overall correlations were lower, certain sphingolipid demonstrated high positive correlations: Cer and dhCer showed very high correlations (r = 0.77 for C22:0 and r = 0.89 for C24:0), and HexCer and LacCer presented high correlations (r = 0.40 for C22:0 and r = 0.50 for C24:0), consistent with the metabolic pathways among sphingolipid classes (Additional file 1: Figs. S3, S4).

Additional file 1: Fig. S5 illustrates the within-class correlation coefficients for SFA ranging from C16:0 to C26:0. In most lipid classes, direct correlations between the immediate precursor fatty acid (shorter chain) and product (longer chain) were observed. In the subsequent analyses with cardiometabolic risk, the neighboring lipids of shorter carbon chain length identified in this network were incorporated into the *precursors-only* adjustment model, and all the neighbors including downstream lipids were incorporated in the *all-class* model.

**Fig. 2:**
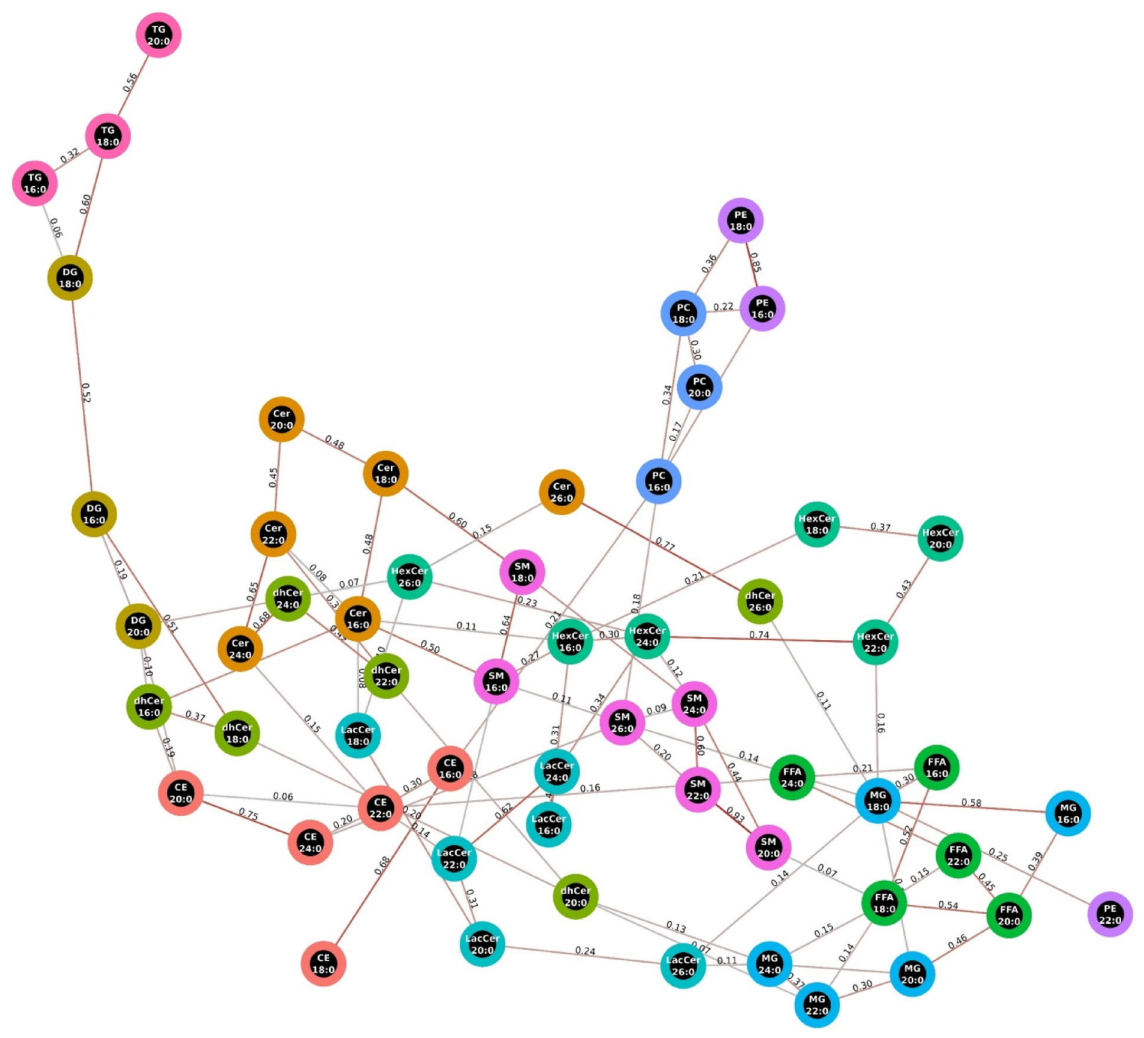
Saturated fatty acids network. Presence of edges represent covariance between two lipids that could not be explained by adjustment for any subset of other lipids and accompanying numerical value reflects the partial Pearson correlation coefficient. Edges with coefficients < 0.05 were removed. CE indicates cholesteryl esters; Cer, ceramides; DG, diacylglycerols; dhCer, dihydroceramides; FFA, free fatty acids; HexCer, hexosylceramides; LacCer, lactosylceramides; MG, monoacylglycerols; PC, phosphatidylcholines; PE, phosphatidylethanolamines; SM, sphingomyelins; TG, and triacylglycerides

Figure 2 presents the data-driven network of all lipids analyzed, including VLCSFA, their precursors C16:0 and C18:0, and the downstream metabolite C26:0. The majority of the connections observed align with known metabolic pathways involved in lipid metabolism, including fatty acid elongation, ceramide desaturation, and the transfer of acyl-CoA groups. In the subsequent analyses with cardiometabolic risk, all the neighboring lipids identified in this network (including both precursors and products) were included in the *all-neighbors* adjustment model.

### Association of VLCSFA with cardiometabolic risk

#### Associations between VLCSFA and CVD risk

**Fig. 3:**
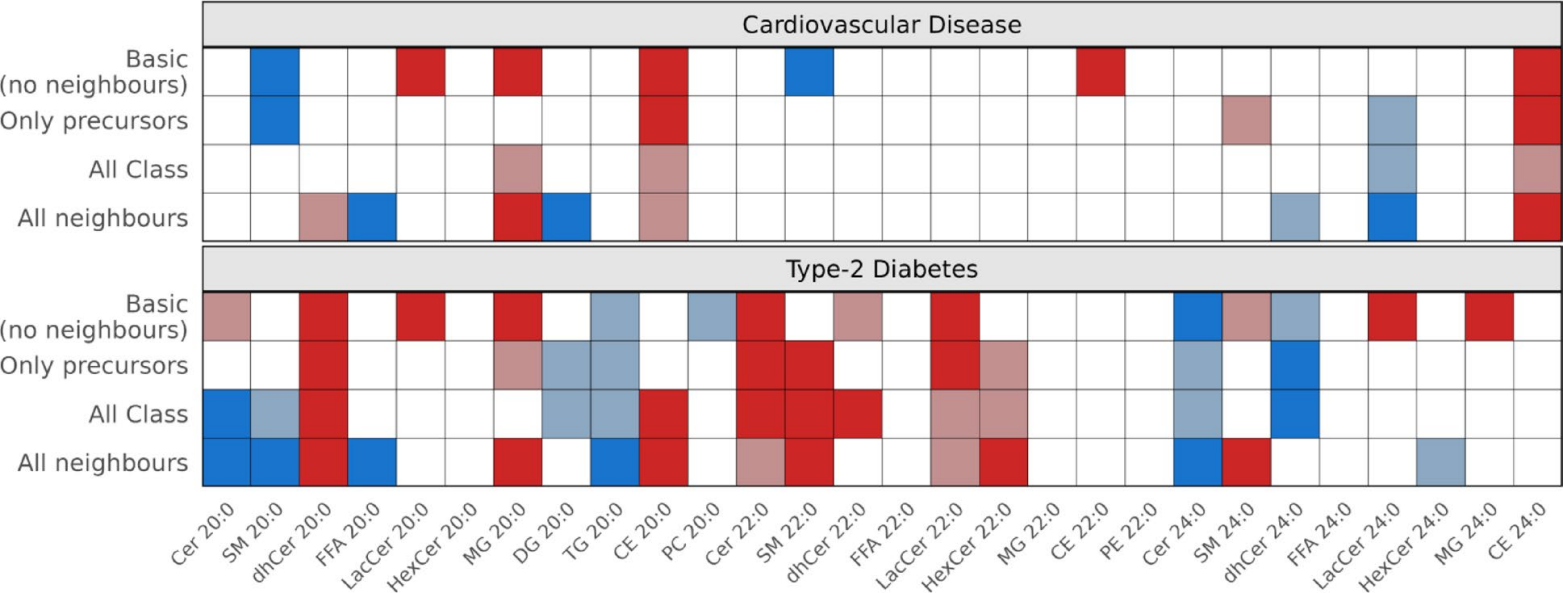
Disease associations of lipids with VLCSFA. Only lipids with significant results in any of the four adjustment models are shown. Frame colors indicate risk associations: blue: lower risk (Hazard Ratio < 1); red: higher risk (Hazard Ratio > 1); white: not significant. Lighter colors indicate nominal significance, darker colors indicate that the association was significant after multiple testing correction using FDR. The basic model was adjusted for age, sex, waist circumference, height, leisure-time physical activity, smoking status (never, former, and current smoker), alcohol intake (none, low [0.1–6 g/d], moderately low [6.1–12.0 g/d], moderately high [12.1–24.0 g/d], high [24.1–60.0 g/d], very high [60.1–96.0 g/d], and extremely high [> 96.1 g/d]), education level (primary school, secondary school/high school and college/higher education degree), fasting status at blood draw (overnight fast, only drink and unfasted), total energy intake, blood pressure (diastolic and systolic), standard clinical blood lipid markers (total cholesterol and triglycerides), antihypertensive medication, lipid-lowering medication, acetylsalicylic acid medication, and the respective sum of the lipid class. Models for incident CVD were additionally adjusted for prevalent T2D and glycated hemoglobin. The precursors-only model was further adjusted for neighboring lipids within the same lipid class with shorter carbon chain length. The class-only model was additionally adjusted for neighboring downstream lipids within the same class. The all-neighbors model was further adjusted for all direct lipid neighbors. *TG and CE were not adjusted for clinical measures of TG and total cholesterol, respectively. CE indicates cholesteryl esters; Cer, ceramides; CVD, cardiovascular disease; DG, diacylglycerols; dhCer, dihydroceramides; FDR, false discovery rate; FFA, free fatty acids; HexCer, hexosylceramides; LacCer, lactosylceramides; MG, monoacylglycerols; PC, phosphatidylcholines; SM, sphingomyelins; TG, triacylglycerides; T2D, type-2 diabetes; and VLCSFA, very long chain saturated fatty acids

Figure 3 shows significant associations between VLCSFA-containing lipids and CVD risk across four adjustment models. In the *basic* model, SM20:0 (HR per 1-SD: 0.63, 95% CI 0.52–0.76) and SM22:0 (0.56, 0.44–0.72) were associated with lower risk. Conversely, CE20-24:0 (1.59, 1.43–1.76, 1.35, 1.14–1.59, and 1.67, 1.48–1.88, respectively), LacCer20:0 (1.29, 1.08–1.52) and MG20:0 (1.24, 1.06–1.45), showed higher risk of CVD. Adjusting for precursors of the same lipid class maintained significance for SM20:0, CE20:0 and CE24:0, but adding downstream within-class lipid metabolites rendered them non-significant. However, in the *all-neighbors* model, the associations were relatively robust or even strengthened for MG20:0 (1.70, 1.28- 2.25) and CE24:0 (1.34, 1.09–1.66). In addition, DG20:0 (0.69, 0.57–0.84), FFA20:0 (0.68, 0.50–0.91), and LacCer24:0 (0.62, 0.46–0.83) were significantly linked to lower risk of CVD; these associations became only visible with adjustment for all neighbor lipids. Full results are shown in Additional file 1: Table S1. No statistically significant (FDR < 0.05) effect measure modification for the association between lipids with VLCSFA and CVD was observed.

#### Associations between VLCSFA and T2D risk

Associations between lipids with VLCSFA and T2D that were significant across any of the four adjustment models are shown in Fig. 3. In the *basic* model, Cer22:0 (HR per SD: 2.03, 95% CI 1.30–3.16)] and dhCer20:0 (1.35, 1.11–1.63) were significantly associated with higher risk of T2D; in contrast, Cer24:0 showed a protective association (0.55, 0.37–0.82). Meanwhile, LacCer20:0 (1.21, 1.04–1.42), LacCer22:0 (1.43, 1.20–1.70) and LacCer24:0 (1.37, 1.12–1.68) were linked to higher risk, as were MG20:0 (1.25, 1.07–1.45) and MG24:0 (1.17, 1.03–1.32). Other lipids, such as TG20:0, PC20:0, and dhCer24:0 also showed associations with lower risk, and Cer20:0 and dhCer22:0, and SM24:0 with higher risk, although not reaching statistical significance after accounting for multiple testing.

Few of these lipid associations with T2D risk were robust against adjustment for correlated lipids within the same class or across classes. Specifically, dhCer20:0 was positively associated across all models (HR *all-neighbors* model: 1.36, 1.07–1.72) while Cer24:0 showed an inverse association (0.46, 0.27–0.79). For Cer20:0, the association was inverted; it was initially linked to higher risk, but shifted to significantly lower risk after adjusting for all neighbors (0.52, 0.35–0.79). Similarly, inverse associations became visible for SM20:0 and FFA20:0 only with adjustments in the *all-class* and *all-neighbors* models, or became stronger for TG20:0. Positive associations for MG20:0 and CE20:0 persisted after precursors adjustment and became stronger and/or significant in the *all-class*/*all-neighbors* models. Regarding C22:0, associations for Cer22:0 and LacCer22:0 were attenuated and not statistically significant after multiple testing correction in the *all-neighbors* model, while SM22:0 and HexCer22:0 showed positive associations after adjustment for precursors and all neighbors, respectively. The latter was also the case for SM24:0. The inverse association of dhCer24:0 strengthened with precursor adjustment but disappeared in the *all-neighbors* model. Other C24:0-containing lipids showed attenuation. Additional file 1: Table S2 details all VLCSFA-lipid associations with T2D risk.

MG20:0 showed a significant interaction with sex in the all the models (p_interaction_ *all-neighbors* model FDR-adjusted = 0.047). The positive association with T2D observed in the overall sample was evident only in men (1.90, 1.31–2.75). FFA20:0 also showed evidence of effect modification by sex: the interaction was significant in the all-class model (p_interaction_ FDR-adjusted = 0.033) but not in the all-neighbors model. This lipid was associated with higher T2D risk in men when adjusted for neighbors within the same lipid class (1.37, 1.08–1.73), but this association was no longer significant after including all direct neighbors (Additional file 1: Table S3).

## Discussion

In this prospective analysis within the EPIC-Potsdam study, we constructed a lipid network based on SFA lipidomics data to identify lipids correlated with individual VLCSFA. Adjusting for neighboring lipids within this network enabled us to distinguish direct associations of individual VLCSFA from associations potentially confounded or mediated by highly correlated lipid species. Accounting for these neighboring lipids revealed that associations with CVD and T2D varied depending on both the fatty acid chain length and the lipid class. Specifically, C22:0 was predominantly linked to higher cardiometabolic risk, whereas relationships for C20:0 and C24:0 were mixed, reflecting variable findings across lipid classes and cardiometabolic outcomes.

Previous studies have highlighted the challenges in assessing the disease risk associated with VLCSFA, because they are highly correlated with their metabolic precursor palmitic acid (C16:0), which is consistently linked with risk factors of cardiometabolic diseases [5]. However, VLCSFA have longer carbon chains that confer different properties compared to their SFA precursors, and these characteristics can vary between different VLCSFA, likely reflecting distinct biochemical and biophysical roles. Another complexity is disentangling their sources; recent studies suggest VLCSFA can serve as biomarkers of peanut intake [20], yet we cannot distinguish those derived from the diet from those produced via elongation of other SFA. The same applies to C16:0, as we cannot separate the contributions of dietary intake and de novo lipogenesis to the pool that is subsequently elongated into VLCSFA.

C24:0 is the predominant VLCSFA in sphingolipids and has unique physical properties, such as modulating membrane fluidity and stability, which enhances cell signaling [4]. Similar benefits on membrane structure and function have not been observed for C22:0 or C20:0, suggesting that these fatty acids do not produce the same effects. We observed that C24:0 is more selectively distributed across lipid classes than C20:0. The synthesis of C24:0 is tightly regulated, as its production through elongation is closely coordinated with its incorporation into sphingolipids [21]. This resulted in very strong correlations between metabolically related lipids like dhCer and Cer. In contrast, C20:0 occurs in a wider range of lipid classes and is primarily incorporated into sphingolipids by ceramide synthase 4 (CerS4). This isoform also incorporates their SFA precursors and is expressed in different tissues compared to CerS2—the main enzyme responsible for C22:0 and C24:0 incorporation [4]. Consistently, associations between C20:0-containing lipids and cardiometabolic diseases varied more with adjustments for correlated lipids.

Using the network approach, we identified specific associations of VLCSFA in different lipid classes with T2D and CVD. Cer are components of cell membranes and function as signaling molecules regulating lipid metabolism [22]. Although their accumulation is often linked to disrupted insulin signaling and β-cell apoptosis—contributing to T2D development [23]- previous studies have yielded conflicting results on Cer with VLCSFA [13, 24]. Generally, Cer20:0 is positively associated with T2D risk when analyses do not adjust for correlated lipids or only adjust for Cer16:0 [24, 25]. However, our study revealed a more complex relationship: the initially detrimental association of Cer20:0 became non-significant and reversed after adjusting for its metabolic precursor Cer18:0. This suggests previous findings may be confounded by the close metabolic relationship between Cer20:0 and Cer18:0, with Cer18:0 playing a more direct role in inflammation and insulin resistance [26, 27]. Interestingly, including Cer22:0 in the model made the Cer20:0 association significantly protective, indicating Cer20:0 might exert protective effects counterbalanced by its role as precursor of Cer22:0. Cer22:0 showed associations with T2D risk across all models. This aligns with other studies linking Cer22:0 to higher plasma insulin levels, and increased insulin resistance and HOMA-IR [24, 28, 29]. Mechanistically, Cer22:0—together with Cer18:0 and Cer24:1—has been implicated in promoting pancreatic β-cell apoptosis, suggesting a pathway through which Cer22:0 contributes to T2D development [30]. In contrast, Cer24:0 was consistently inversely associated with T2D (~ 54% lower hazard in the all-neighbors model), opposite to the positive associations reported for precursor SFA such as C16:0 in meta‑analyses (relative risks up to ~ 1.53) [31]. This finding aligns with previous murine studies showing different roles of ceramide species, where precursor Cer like Cer16:0 and Cer18:0 promote insulin resistance, while Cer24:0 does not appear to exert such effects [32]. However, while Cer24:0 has been used as the denominator in risk ratios with precursor Cer to improve prediction of CVD due to its protective associations [33], we did not observe any significant relationship between Cer24:0 and CVD risk.

A similarly diverse pattern was observed for SM: SM20:0 was associated with a lower risk of T2D, whereas SM24:0 was linked to a higher risk, only after adjusting for downstream correlated lipids. In contrast, SM22:0 showed a risk association after accounting for the precursor SM20:0, suggesting that this relationship had been masked by confounding from the correlated precursor. This is consistent with our findings in other lipid classes, where C22:0–containing species tend to be associated with higher risk. Previous studies have not reported significant associations between development of T2D or its markers and SM containing VLCSFA [25, 28], likely because they did not adjust for correlated lipids; this aligns with our *basic model* results, as the associations only became evident after adjustment for neighbors. Mechanistically, the chain length of sphingomyelin influences membrane packing: longer chains (≥ C22) exhibit stronger condensation with cholesterol and stabilize more ordered domains [34]. Despite limited evidence in humans linking SM24:0 explicitly to T2D, preclinical studies show that SM24:0—but not Cer24:0—can activate macrophage inflammatory markers, suggesting a structure-dependent role of sphingolipids with C24:0 in modulating inflammation relevant to T2D [35]. In contrast with these findings, higher concentrations of SM species containing VLCSFA have been associated with lower risk of incident atrial fibrillation and heart failure [36, 37]. However, these studies only adjusted for SM16:0, which, according to our network, was not directly connected to the VLCSFA-containing SM species. Still consistent with this, in our data, SM20:0 and SM22:0 were initially protective against CVD. However, while SM20:0 remained inversely associated when adjusted for precursors, the association for SM22:0 was attenuated with such adjustment. This suggests that, as in T2D, the apparent relationship between SM22:0 and CVD in the *basic model* was largely explained by confounding by SM20:0. Nevertheless, the inverse association of SM20:0 was slightly attenuated but lost precision after adjusting for downstream and other neighboring lipids, likely as a consequence of mutual adjustment of strongly correlated lipids.

As expected given their metabolic relationship, dhCer and Cer species with VLCSFA were strongly correlated; however, their associations with clinical outcomes differed from those of Cer. dhCer20:0 was consistently associated with higher T2D risk across all adjustment models and showed nominal significance for higher CVD risk in the *all-neighbors* model. dhCer22:0 was also linked to higher T2D risk, but it lost significance after including Cer22:0 in the model, suggesting that the ceramide may be driving the relationship. Similarly, dhCer24:0 did not retain a protective association with T2D once Cer24:0 was included in the model, indicating that the association was likely influenced by the downstream ceramide. This aligns with previous literature reporting associations between higher dhCer levels and diabetes markers and linking cellular dhCer administration to decreases insulin sensitivity [38, 39]. However, most studies have not analyzed individual dhCer species. Further research is needed to understand the role of dhCer containing different SFA on cardiometabolic health.

Research on glycosphingolipids, particularly HexCer and LacCer, in T2D and CVD reveals complex and sometimes contradictory findings. Preclinical studies showed that glucosylated Cer impair insulin signaling [40], thereby promoting T2D development. However, human studies have generally overlooked HexCer with VLCSFA, and one found no link between HexCer22:0 and HexCer24:0 with T2D [27], matching our findings in the basic adjustment models. Adjusting for precursor lipids revealed a significant risk association between HexCer22:0 and T2D, a relationship that was likely previously hidden by other HexCer species, and was strengthened by including downstream lipids. Conversely, HexCer24:0 showed a protective association, which may have been masked before by its correlation with SM24:0. Evidence on LacCer is also mixed. A study linked LacCer accumulation to higher inflammation and production of reactive oxygen species [41], and this finding was supported in human data showing an association between LacCer containing VLCSFA and higher CVD [42]. However, others reported inverse associations with T2D for LacCer16:0, LacCer18:0, LacCer24:1 [29]. We found consistent higher T2D risk with LacCer22:0. In contrast, LacCer20:0 and LacCer24:0 risk associations varied with neighboring lipids; no association was observed after accounting for LacCer18:0 and LacCer16:0, respectively, suggesting confounding by precursor lipids. We also observed a novel association of LacCer24:0 and a 38% lower risk of CVD, one of the strongest inverse associations in the all-neighbors model. Although circulating levels are low, several lipid mediators act effectively at micromolar concentrations, supporting the biological plausibility of this finding [43].

Surprisingly, FFA20:0 showed inverse associations with T2D and CVD after adjusting for all neighboring lipids, likely masked in the *basic model* by correlation with the detrimental MG16:0 [17], a confounding particularly relevant for men. Among acylglycerides, TG20:0 and DG20:0 were linked to lower risks of CVD and T2D, respectively, contrasting with the associations observed for other lipids of these classes [17]. Since certain FFA and acyl-CoA derivates activate peroxisome proliferator activated receptor alpha (PPARα)—a key regulator of lipid metabolism, inflammation, and glucose balance—we hypothesize these molecular species influence cardiometabolic health through PPARα modulation [44]. As expected, CE were associated with higher risk of both T2D and CVD; their link to CVD may reflect their role in atherosclerotic plaque formation, whereas in T2D, the association may be explained by the elevated levels of very low-density lipoprotein (VLDL) that typically accompany the pre-clinical phase of the disease [45, 46].

Our study has several strengths. By constructing the lipid network and incorporating neighboring lipids into the Cox models, we could account for confounding by precursor lipids and separate direct associations of individual VLCSFA from those of correlated, potentially downstream lipids. We used comprehensive lipidomics data from a large, well-characterized prospective cohort with incident T2D and CVD outcomes. To our knowledge, this is the first prospective study to assess VLCSFA with cardiometabolic risk across a large range of lipid classes. Our study also has limitations. To isolate lipid-specific associations, we conditioned on highly correlated neighbors within a data‑driven lipid network and adjusted for lipid class sums. While this improves interpretability, it can introduce collinearity and reduce precision. The same consideration applies to our adjustment for total lipid class sums. Furthermore, we did not explicitly model interaction terms among VLCSFA-containing lipids. our analytical approach assumed independent biological effects of individual fatty acid species, which may not reflect the complex metabolic interrelationships that exist in vivo.

## Conclusions

In conclusion, we developed a data-driven correlation network of plasma lipids containing the VLCSFA C20:0, C22:0, and C24:0. Integrating this network to incident cardiometabolic outcomes showed that these fatty acids are associated differently with cardiometabolic risk depending on the lipid class, with C22:0 particularly linked to higher risk across several lipid classes. Importantly, many of these associations only became apparent after adjusting for neighboring lipids, highlighting the importance of accounting for lipid interrelationships. These results challenge the conventional view of SFA as uniformly detrimental and emphasize the need to develop standardized, class-specific VLCSFA analyses, accounting for correlated lipids.

## Supplementary Information

Below is the link to the electronic supplementary material.

Supplementary Material 1

## Data Availability

The data sets analyzed during the current study are not publicly available due to data protection regulations. In accordance with German federal and state data protection regulations, epidemiological data analyses of EPIC-Potsdam may be initiated upon an informal inquiry addressed to the secretariat of the Human Study Center (office.hsz@dife.de). Each request will then have to pass a formal process of application and review by the respective principal investigator and a scientific board.

## Abbreviations

CVD: Cardiovascular disease
CE: Cholesteryl esters
Cer: Ceramides
Cers: Ceramide synthase
DG: Diacylglycerols
dhCer: Dihydroceramides
FDR: False discovery rate
FFA: Free fatty acids
HexCer: Hexosylceramides
LacCer: Lactosylceramides
LPC: Lysophosphatidylcholines
LPE: Lysophosphatidylethanolamines
MG: Monoacylglycerols
MRM: Multiple reaction monitoring
PPARα: Peroxisome proliferator activated receptor alpha
PC: Phosphatidylcholines
PE: Phosphatidylethanolamines
PEOs: Phosphatidylethanolamine ethers
PEPs: Phosphatidylethanolamine plasmalogens
PI: Phosphatidylinositol
SFA: Saturated fatty acids
SM: Sphingomyelins
TG: Triacylglycerols
T2D: Type 2 diabetes
VLCSFA: Very long-chain saturated fatty acids
VLDL: Very low-density lipoprotein
