# Supplementary material for: Very long-chain saturated fatty acids in plasma lipids: association with cardiometabolic risk influenced by lipid interactions
Source: Cardiovasc Diabetol. 2025 Dec 22;25:24. doi: 10.1186/s12933-025-03037-4 (PMC12838474; doi:10.1186/s12933-025-03037-4)
Supplement: Supplementary file 1 — Supplementary Material 1 [file 12933_2025_3037_MOESM1_ESM.docx]

**Very Long-Chain Saturated Fatty Acids in Plasma Lipids: Association with Cardiometabolic Risk Influenced by Lipid Interactions**

**Additional File 1.**

**Expanded Methods.** Detailed Methods.

**Supplementary Table 1.** Prospective associations of VLCSFA lipid species (per 1-SD) with CVD.

**Supplementary Table 2.** Prospective associations of VLCSFA lipid species (per 1-SD) with T2D.

**Supplementary Table 3.** Sex-specific prospective associations of VLCSFA lipid species (per 1-SD) with T2D for lipids with significant sex interactions.

**Supplementary Figure 1.** Flow-chart of the T2D and CVD case-cohort design.

**Supplementary Figure 2.** Spearman rank correlation coefficients for C20:0 across lipid species.

**Supplementary Figure 3** Spearman rank correlation coefficients for C22:0 across lipid species.

**Supplementary Figure 4.** Spearman rank correlation coefficients for C24:0 across lipid species.

**Supplementary Figure 5.** Within class correlations (lipids from the same class with different saturated fatty acid).

**Expanded Methods**

Lipids were extracted in methanol:dichloromethane, concentrated under nitrogen and reconstituted in ammonium acetate dichloromethane:methanol. A SelexION® -5500 QTRAP mass spectrometer operated in multiple reaction monitoring (MRM) mode and positive/negative switching was used. The lipid extracts were infused into the ionization source and separated by differential mobility spectrometry. Lipid mass and characteristic fragments were determined using 1,100 MRM transitions and 50 isotopically labeled internal standards that were simultaneously introduced with the biological sample. Molecular species were quantified by taking the ratio of the signal intensity of each target compound to that of its assigned internal standard, then multiplying by the concentration of internal standards included in the sample.

To assess stability during long-term storage, we compared median concentrations of all assayed lipids in baseline plasma (1994–1998) with plasma collected for a pilot study in 2014–2016 (n=35), analyzing both sets concurrently in 2016. Median levels were comparable, providing no evidence that prolonged storage altered lipid concentrations.

**Supplementary Tables**

| **Table S1.** Prospective associations of VLCSFA lipid species (per 1-SD) with CVD. | | | | |  |
| --- | --- | --- | --- | --- | --- |
|  | Model | | HR (95% CI) | *p*-value |  |
| C20:0 |  | |  |  |  |
| Ceramide C20:0 | Basic | | 1.02 (0.77; 1.34) | 0.892 |  |
|  | Only-precursors | | 0.69 (0.46; 1.04) | 0.074 |  |
|  | All-class | | 0.72 (0.47; 1.12) | 0.148 |  |
|  | All-neighbors | | 0.72 (0.47; 1.12) | 0.148 |  |
| Sphingomyelin C20:0 | Basic | | 0.63 (0.52; 0.76) | <0.001 |  |
|  | Only-precursors | | 0.63 (0.52; 0.76) | <0.001 |  |
|  | All-class | | 0.74 (0.51; 1.09) | 0.130 |  |
|  | All-neighbors | | 0.75 (0.48; 1.16) | 0.191 |  |
| Dihydroceramide C20:0 | Basic | | 1.17 (0.96; 1.44) | 0.127 |  |
|  | Only-precursors | | 1.23 (1.00; 1.52) | 0.053 |  |
|  | All-class | | 1.20 (0.96; 1.50) | 0.111 |  |
|  | All-neighbors | | 1.34 (1.05; 1.70) | 0.019 |  |
| Free Fatty Acid C20:0 | Basic | | 1.13 (0.98; 1.30) | 0.094 |  |
|  | Only-precursors | | 1.13 (0.95; 1.34) | 0.160 |  |
|  | All-class | | 1.11 (0.92; 1.32) | 0.278 |  |
|  | All-neighbors | | 0.68 (0.50; 0.91) | 0.009 |  |
| Hexosylceramide C20:0 | Basic | | 0.93 (0.70; 1.24) | 0.621 |  |
|  | Only-precursors | | 0.81 (0.59; 1.12) | 0.203 |  |
|  | All-class | | 0.82 (0.59; 1.13) | 0.219 |  |
|  | All-neighbors | | 0.82 (0.59; 1.13) | 0.219 |  |
| Lactosylceramide C20:0 | Basic | | 1.29 (1.08; 1.52) | 0.004 |  |
|  | Only-precursors | | 1.11 (0.90; 1.36) | 0.340 |  |
|  | All-class | | 1.00 (0.79; 1.27) | 0.996 |  |
|  | All-neighbors | | 1.00 (0.79; 1.27) | 0.996 |  |
| Monoglyceride C20:0 | Basic | | 1.24 (1.06; 1.45) | 0.008 |  |
|  | Only-precursors | | 1.19 (1.00; 1.41) | 0.053 |  |
|  | All-class | | 1.46 (1.14; 1.88) | 0.003 |  |
|  | All-neighbors | | 1.70 (1.28; 2.25) | <0.001 |  |
| Diglyceride C20:0 | Basic | | 0.98 (0.84; 1.14) | 0.762 |  |
|  | Only-precursors | | 0.90 (0.77; 1.06) | 0.220 |  |
|  | All-class | | 0.90 (0.77; 1.06) | 0.220 |  |
|  | All-neighbors | | 0.69 (0.57; 0.84) | <0.001 |  |
| Triglyceride C20:0 | Basic | | 1.17 (0.84; 1.62) | 0.350 |  |
|  | Only-precursors | | 0.97 (0.67; 1.41) | 0.883 |  |
|  | All-class | | 0.97 (0.67; 1.41) | 0.883 |  |
|  | All-neighbors | | 0.97 (0.67; 1.41) | 0.883 |  |
| Cholesteryl ester C20:0 | Basic | | 1.59 (1.43; 1.76) | <0.001 |  |
|  | Only-precursors | | 1.59 (1.43; 1.76) | <0.001 |  |
|  | All-class | | 1.23 (1.02; 1.48) | 0.027 |  |
|  | All-neighbors | | 1.33 (1.06; 1.68) | 0.015 |  |
| Phosphatidylcholine C20:0 | Basic | | 0.89 (0.77; 1.04) | 0.138 |  |
|  | Only-precursors | | 0.88 (0.75; 1.03) | 0.116 |  |
|  | All-class | | 0.88 (0.75; 1.03) | 0.116 |  |
|  | All-neighbors | | 0.88 (0.75; 1.03) | 0.116 |  |
| C22:0 | |  |  |  | |
| Ceramide C22:0 | | Basic | 0.78 (0.50; 1.22) | 0.278 | |
|  |  | Only-precursors | 0.75 (0.47; 1.20) | 0.238 | |
|  |  | All-class | 0.75 (0.47; 1.21) | 0.240 | |
|  |  | All-neighbors | 0.75 (0.44; 1.28) | 0.297 | |
| Sphingomyelin C22:0 | | Basic | 0.56 (0.44; 0.72) | <0.001 | |
|  |  | Only-precursors | 0.87 (0.53; 1.41) | 0.566 | |
|  |  | All-class | 0.70 (0.42; 1.17) | 0.177 | |
|  |  | All-neighbors | 0.70 (0.42; 1.17) | 0.177 | |
| Dihydroceramide C22:0 | | Basic | 1.20 (0.95; 1.52) | 0.118 | |
|  |  | Only-precursors | 1.18 (0.92; 1.50) | 0.186 | |
|  |  | All-class | 1.21 (0.91; 1.60) | 0.184 | |
|  |  | All-neighbors | 1.25 (0.89; 1.76) | 0.196 | |
| Free Fatty Acid C22:0 | | Basic | 1.12 (0.97; 1.28) | 0.118 | |
|  |  | Only-precursors | 1.07 (0.91; 1.26) | 0.396 | |
|  |  | All-class | 1.01 (0.83; 1.22) | 0.940 | |
|  |  | All-neighbors | 1.01 (0.83; 1.22) | 0.940 | |
| Hexosylceramide C22:0 | | Basic | 0.84 (0.56; 1.25) | 0.384 | |
|  |  | Only-precursors | 0.84 (0.56; 1.27) | 0.415 | |
|  |  | All-class | 0.84 (0.56; 1.26) | 0.407 | |
|  |  | All-neighbors | 0.96 (0.63; 1.46) | 0.856 | |
| Lactosylceramide C22:0 | | Basic | 1.13 (0.92; 1.39) | 0.245 | |
|  |  | Only-precursors | 1.02 (0.82; 1.27) | 0.837 | |
|  |  | All-class | 1.28 (0.98; 1.68) | 0.073 | |
|  |  | All-neighbors | 1.10 (0.83; 1.45) | 0.503 | |
| Monoglyceride C22:0 | | Basic | 1.06 (0.93; 1.21) | 0.403 | |
|  |  | Only-precursors | 0.84 (0.69; 1.01) | 0.067 | |
|  |  | All-class | 0.92 (0.72; 1.17) | 0.491 | |
|  |  | All-neighbors | 0.87 (0.68; 1.12) | 0.275 | |
| Cholesteryl ester C22:0 | | Basic | 1.35 (1.14; 1.59) | <0.001 | |
|  |  | Only-precursors | 1.10 (0.92; 1.32) | 0.295 | |
|  |  | All-class | 1.03 (0.86; 1.24) | 0.737 | |
|  |  | All-neighbors | 1.02 (0.84; 1.25) | 0.815 | |
| Phosphatidylethanolamine C22:0 | | Basic | 0.98 (0.87; 1.12) | 0.813 | |
|  |  | Only-precursors | 0.98 (0.87; 1.12) | 0.813 | |
|  |  | All-class | 0.98 (0.87; 1.12) | 0.813 | |
|  |  | All-neighbors | 0.88 (0.77; 1.01) | 0.067 | |
| C24:0 | |  |  |  | |
| Ceramide C24:0 | | Basic | 0.83 (0.53; 1.32) | 0.441 | |
|  |  | Only-precursors | 0.79 (0.50; 1.26) | 0.325 | |
|  |  | All-class | 0.79 (0.50; 1.26) | 0.325 | |
|  |  | All-neighbors | 1.00 (0.53; 1.87) | 0.999 | |
| Sphingomyelin C24:0 | | Basic | 1.32 (0.99; 1.75) | 0.060 | |
|  |  | Only-precursors | 1.36 (1.02; 1.81) | 0.036 | |
|  |  | All-class | 1.28 (0.95; 1.71) | 0.100 | |
|  |  | All-neighbors | 1.28 (0.91; 1.80) | 0.151 | |
| Dihydroceramide C24:0 | | Basic | 1.00 (0.81; 1.23) | 0.997 | |
|  |  | Only-precursors | 0.91 (0.72; 1.15) | 0.418 | |
|  |  | All-class | 0.91 (0.72; 1.15) | 0.418 | |
|  |  | All-neighbors | 0.62 (0.39; 0.98) | 0.042 | |
| Free Fatty Acid C24:0 | | Basic | 1.12 (0.98; 1.28) | 0.109 | |
|  |  | Only-precursors | 1.14 (0.98; 1.33) | 0.100 | |
|  |  | All-class | 1.14 (0.98; 1.33) | 0.100 | |
|  |  | All-neighbors | 1.07 (0.90; 1.26) | 0.449 | |
| Hexosylceramide C24:0 | | Basic | 1.21 (0.80; 1.83) | 0.362 | |
|  |  | Only-precursors | 1.42 (0.90; 2.24) | 0.127 | |
|  |  | All-class | 1.33 (0.83; 2.13) | 0.228 | |
|  |  | All-neighbors | 1.39 (0.80; 2.44) | 0.246 | |
| Lactosylceramide C24:0 | | Basic | 0.85 (0.68; 1.05) | 0.132 | |
|  |  | Only-precursors | 0.71 (0.54; 0.92) | 0.010 | |
|  |  | All-class | 0.71 (0.54; 0.92) | 0.010 | |
|  |  | All-neighbors | 0.62 (0.46; 0.83) | 0.001 | |
| Monoglyceride C24:0 | | Basic | 1.04 (0.91; 1.19) | 0.540 | |
|  |  | Only-precursors | 0.87 (0.69; 1.10) | 0.249 | |
|  |  | All-class | 0.87 (0.69; 1.10) | 0.249 | |
|  |  | All-neighbors | 0.80 (0.62; 1.02) | 0.073 | |
| Cholesteryl ester C24:0 | | Basic | 1.67 (1.48; 1.88) | <0.001 | |
|  |  | Only-precursors | 1.37 (1.11; 1.69) | 0.004 | |
|  |  | All-class | 1.37 (1.11; 1.69) | 0.004 | |
|  |  | All-neighbors | 1.34 (1.09; 1.66) | 0.006 | |
| VLCSFA, very long chain saturated fatty acids; CVD, cardiovascular disease; HR, hazard ratio; CI, confidence interval.  The *basic* model was adjusted for age, sex, waist circumference, height, leisure-time physical activity, smoking status, alcohol intake, education level, fasting status at blood draw, total energy intake, blood pressure (diastolic and systolic), standard clinical blood lipid markers (total cholesterol and triglycerides), antihypertensive medication, lipid-lowering medication, acetylsalicylic acid medication, prevalent diabetes, glycated hemoglobin, and the respective sum of the lipid class. The *precursors-only* model was additionally adjusted for neighboring precursor lipids (shorter carbon chain length) within the same lipid class. The *all-class* model was further adjusted for all lipid neighbors within the same class. The *all-neighbors* model was adjusted for all direct neighbors.  Triglycerides and cholesteryl esters were not adjusted for clinical measures of triglycerides and total cholesterol, respectively. | | | | | |

| **Table S2.** Prospective associations of VLCSFA lipid species (per 1-SD) with T2D. | | | | | | |  |
| --- | --- | --- | --- | --- | --- | --- | --- |
|  | Model | HR (95% CI) | | p-value | | |  |
| C20:0 |  |  | |  | | |  |
| Ceramide C20:0 | Basic | 1.36 (1.05; 1.77) | | 0.022 | | |  |
|  | Only-precursors | 0.77 (0.52; 1.15) | | 0.199 | | |  |
|  | All-class | 0.52 (0.35; 0.79) | | 0.002 | | |  |
|  | All-neighbors | 0.52 (0.35; 0.79) | | 0.002 | | |  |
| Sphingomyelin C20:0 | Basic | 1.01 (0.84; 1.22) | | 0.889 | | |  |
|  | Only-precursors | 1.01 (0.84; 1.22) | | 0.889 | | |  |
|  | All-class | 0.64 (0.45; 0.93) | | 0.017 | | |  |
|  | All-neighbors | 0.61 (0.40; 0.91) | | 0.016 | | |  |
| Dihydroceramide C20:0 | Basic | 1.35 (1.11; 1.63) | | 0.003 | | |  |
|  | Only-precursors | 1.36 (1.11; 1.67) | | 0.003 | | |  |
|  | All-class | 1.32 (1.07; 1.63) | | 0.009 | | |  |
|  | All-neighbors | 1.36 (1.07; 1.72) | | 0.011 | | |  |
| Free Fatty Acid C20:0 | Basic | 1.06 (0.92; 1.23) | | 0.413* | | |  |
|  | Only-precursors | 1.08 (0.91; 1.27) | | 0.387* | | |  |
|  | All-class | 1.11 (0.93; 1.33) | | 0.257* | | |  |
|  | All-neighbors | 0.69 (0.51; 0.94) | | 0.018 | | |  |
| Hexosylceramide C20:0 | Basic | 0.92 (0.70; 1.21) | | 0.545 | | |  |
|  | Only-precursors | 0.92 (0.67; 1.25) | | 0.583 | | |  |
|  | All-class | 0.78 (0.56; 1.07) | | 0.118 | | |  |
|  | All-neighbors | 0.78 (0.56; 1.07) | | 0.118 | | |  |
| Lactosylceramide C20:0 | Basic | 1.21 (1.04; 1.42) | | 0.014 | | |  |
|  | Only-precursors | 1.15 (0.95; 1.39) | | 0.145 | | |  |
|  | All-class | 0.93 (0.75; 1.15) | | 0.488 | | |  |
|  | All-neighbors | 0.93 (0.75; 1.15) | | 0.488 | | |  |
| Monoglyceride C20:0 | Basic | 1.25 (1.07; 1.45) | | 0.005* | | |  |
|  | Only-precursors | 1.25 (1.05; 1.50) | | 0.012* | | |  |
|  | All-class | 1.25 (0.97; 1.61) | | 0.087* | | |  |
|  | All-neighbors | 1.49 (1.12; 1.99) | | 0.006* | | |  |
| Diglyceride C20:0 | Basic | 0.94 (0.80; 1.11) | | 0.467 | | |  |
|  | Only-precursors | 0.83 (0.70; 0.99) | | 0.035 | | |  |
|  | All-class | 0.83 (0.70; 0.99) | | 0.035 | | |  |
|  | All-neighbors | 0.82 (0.67; 1.00) | | 0.053 | | |  |
| Triglyceride C20:0 | Basic | 0.73 (0.53; 1.00) | | 0.049 | | |  |
|  | Only-precursors | 0.65 (0.45; 0.93) | | 0.018 | | |  |
|  | All-class | 0.65 (0.45; 0.93) | | 0.018 | | |  |
|  | All-neighbors | 0.65 (0.45; 0.93) | | 0.018 | | |  |
| Cholesteryl ester C20:0 | Basic | 1.13 (0.97; 1.31) | | 0.112 | | |  |
|  | Only-precursors | 1.13 (0.97; 1.31) | | 0.112 | | |  |
|  | All-class | 1.29 (1.07; 1.56) | | 0.007 | | |  |
|  | All-neighbors | 1.39 (1.08; 1.80) | | 0.011 | | |  |
| Phosphatidylcholine C20:0 | Basic | 0.86 (0.75; 0.99) | | 0.032 | | |  |
|  | Only-precursors | 0.90 (0.77; 1.05) | | 0.183 | | |  |
|  | All-class | 0.90 (0.77; 1.05) | | 0.183 | | |  |
|  | All-neighbors | 0.90 (0.77; 1.05) | | 0.183 | | |  |
| C22:0 |  | |  | |  | | |
| Ceramide C22:0 | Basic | | 2.03 (1.30; 3.16) | | 0.002 | | |
|  | Only-precursors | | 1.84 (1.18; 2.89) | | 0.008 | | |
|  | All-class | | 1.79 (1.15; 2.79) | | 0.010 | | |
|  | All-neighbors | | 1.69 (1.02; 2.78) | | 0.040 | | |
| Sphingomyelin C22:0 | Basic | | 1.25 (0.98; 1.58) | | 0.071 | | |
|  | Only-precursors | | 2.19 (1.38; 3.46) | | 0.001 | | |
|  | All-class | | 2.02 (1.25; 3.26) | | 0.004 | | |
|  | All-neighbors | | 2.02 (1.25; 3.26) | | 0.004 | | |
| Dihydroceramide C22:0 | Basic | | 1.26 (1.01; 1.57) | | 0.043 | | |
|  | Only-precursors | | 1.21 (0.96; 1.52) | | 0.103 | | |
|  | All-class | | 1.45 (1.11; 1.90) | | 0.007 | | |
|  | All-neighbors | | 1.21 (0.88; 1.67) | | 0.241 | | |
| Free Fatty Acid C22:0 | Basic | | 0.98 (0.85; 1.12) | | 0.738 | | |
|  | Only-precursors | | 0.93 (0.79; 1.10) | | 0.416 | | |
|  | All-class | | 0.91 (0.76; 1.10) | | 0.351 | | |
|  | All-neighbors | | 0.91 (0.76; 1.10) | | 0.351 | | |
| Hexosylceramide C22:0 | Basic | | 1.47 (1.00; 2.17) | | 0.051 | | |
|  | Only-precursors | | 1.55 (1.06; 2.28) | | 0.024 | | |
|  | All-class | | 1.56 (1.07; 2.29) | | 0.022 | | |
|  | All-neighbors | | 1.73 (1.17; 2.56) | | 0.006 | | |
| Lactosylceramide C22:0 | Basic | | 1.43 (1.20; 1.70) | | <0.001 | | |
|  | Only-precursors | | 1.38 (1.14; 1.66) | | 0.001 | | |
|  | All-class | | 1.27 (1.01; 1.60) | | 0.045 | | |
|  | All-neighbors | | 1.33 (1.04; 1.70) | | 0.024 | | |
| Monoglyceride C22:0 | Basic | | 1.11 (0.98; 1.25) | | 0.097 | | |
|  | Only-precursors | | 0.95 (0.79; 1.14) | | 0.570 | | |
|  | All-class | | 0.86 (0.69; 1.08) | | 0.200 | | |
|  | All-neighbors | | 0.87 (0.68; 1.10) | | 0.235 | | |
| Cholesteryl ester C22:0 | Basic | | 0.90 (0.78; 1.04) | | 0.143 | | |
|  | Only-precursors | | 0.87 (0.75; 1.02) | | 0.090 | | |
|  | All-class | | 0.92 (0.78; 1.08) | | 0.299 | | |
|  | All-neighbors | | 0.92 (0.77; 1.11) | | 0.377 | | |
| Phosphatidylethanolamine C22:0 | Basic | | 0.99 (0.88; 1.12) | | 0.862 | | |
|  | Only-precursors | | 0.99 (0.88; 1.12) | | 0.862 | | |
|  | All-class | | 0.99 (0.88; 1.12) | | 0.862 | | |
|  | All-neighbors | | 0.93 (0.82; 1.06) | | 0.301 | | |
| C24:0 |  | |  | |  |  |  |
| Ceramide C24:0 | Basic | | 0.55 (0.37; 0.82) | | 0.004 |  |  |
|  | Only-precursors | | 0.61 (0.41; 0.92) | | 0.019 |  |  |
|  | All-class | | 0.61 (0.41; 0.92) | | 0.019 |  |  |
|  | All-neighbors | | 0.46 (0.27; 0.79) | | 0.005 |  |  |
| Sphingomyelin C24:0 | Basic | | 1.36 (1.05; 1.77) | | 0.019 |  |  |
|  | Only-precursors | | 1.25 (0.95; 1.65) | | 0.114 |  |  |
|  | All-class | | 1.25 (0.94; 1.66) | | 0.121 |  |  |
|  | All-neighbors | | 1.61 (1.15; 2.26) | | 0.005 |  |  |
| Dihydroceramide C24:0 | Basic | | 0.82 (0.70; 0.98) | | 0.025 |  |  |
|  | Only-precursors | | 0.68 (0.55; 0.84) | | <0.001 |  |  |
|  | All-class | | 0.68 (0.55; 0.84) | | <0.001 |  |  |
|  | All-neighbors | | 1.18 (0.80; 1.74) | | 0.401 |  |  |
| Free Fatty Acid C24:0 | Basic | | 1.01 (0.88; 1.16) | | 0.858 |  |  |
|  | Only-precursors | | 1.03 (0.88; 1.20) | | 0.688 |  |  |
|  | All-class | | 1.03 (0.88; 1.20) | | 0.688 |  |  |
|  | All-neighbors | | 1.11 (0.94; 1.31) | | 0.232 |  |  |
| Hexosylceramide C24:0 | Basic | | 0.84 (0.58; 1.20) | | 0.325 |  |  |
|  | Only-precursors | | 1.04 (0.70; 1.55) | | 0.837 |  |  |
|  | All-class | | 1.09 (0.72; 1.64) | | 0.689 |  |  |
|  | All-neighbors | | 0.56 (0.33; 0.92) | | 0.023 |  |  |
| Lactosylceramide C24:0 | Basic | | 1.37 (1.12; 1.68) | | 0.002 |  |  |
|  | Only-precursors | | 1.16 (0.91; 1.49) | | 0.239 |  |  |
|  | All-class | | 1.16 (0.91; 1.49) | | 0.239 |  |  |
|  | All-neighbors | | 1.31 (1.00; 1.73) | | 0.053 |  |  |
| Monoglyceride C24:0 | Basic | | 1.17 (1.03; 1.32) | | 0.012 |  |  |
|  | Only-precursors | | 1.16 (0.92; 1.46) | | 0.203 |  |  |
|  | All-class | | 1.16 (0.92; 1.46) | | 0.203 |  |  |
|  | All-neighbors | | 1.13 (0.88; 1.44) | | 0.341 |  |  |
| Cholesteryl ester C24:0 | Basic | | 0.96 (0.80; 1.14) | | 0.627 |  |  |
|  | Only-precursors | | 0.83 (0.65; 1.06) | | 0.134 |  |  |
|  | All-class | | 0.83 (0.65; 1.06) | | 0.134 |  |  |
|  | All-neighbors | | 0.79 (0.62; 1.00) | | 0.054 |  |  |
| VLCSFA, very long chain saturated fatty acids; T2D, type-2 diabetes; HR, hazard ratio; CI, confidence interval.  The *basic* model was adjusted for age, sex, waist circumference, height, leisure-time physical activity, smoking status, alcohol intake, education level, fasting status at blood draw, total energy intake, blood pressure (diastolic and systolic), standard clinical blood lipid markers (total cholesterol and triglycerides), antihypertensive medication, lipid-lowering medication, acetylsalicylic acid medication, and the respective sum of the lipid class. The *only-precursors* model was additionally adjusted for neighboring precursor lipids (shorter carbon chain length) within the same lipid class. The *all-class* model was further adjusted for all lipid neighbors within the same class. The *all-neighbors* model was adjusted for all direct neighbors.  Triglycerides and cholesteryl esters were not adjusted for clinical measures of triglycerides and total cholesterol, respectively.  (*) Indicates a statistically significant (FDR<0.05) interaction with sex. | | | | | |  |  |

| **Table S3.** Sex-specific prospective associations of VLCSFA lipid species (per 1-SD) with T2D for lipids with significant sex interactions. | | | |
| --- | --- | --- | --- |
| C20:0 | Adjustment Model | HR (95% CI) | p-value |
| *Men* |  |  |  |
| Free Fatty Acid C20:0 | Basic | 1.31 (1.08; 1.59) | 0.006 |
|  | Only-precursors | 1.33 (1.06; 1.65) | 0.012 |
|  | All-class | 1.37 (1.08; 1.73) | 0.008 |
|  | All-neighbors | 0.76 (0.52; 1.10) | 0.147 |
| Monoglyceride C20:0 | Basic | 1.47 (1.20; 1.80) | <0.001 |
|  | Only-precursors | 1.55 (1.23; 1.94) | <0.001 |
|  | All-class | 1.67 (1.19; 2.33) | 0.003 |
|  | All-neighbors | 1.90 (1.31; 2.75) | 0.001 |
| *Women* |  |  |  |
| Free Fatty Acid C20:0 | Basic | 0.86 (0.71; 1.04) | 0.117 |
|  | Only-precursors | 0.90 (0.72; 1.12) | 0.342 |
|  | All-class | 0.88 (0.69; 1.12) | 0.283 |
|  | All-neighbors | 0.54 (0.35; 0.82) | 0.004 |
| Monoglyceride C20:0 | Basic | 0.96 (0.77; 1.20) | 0.735 |
|  | Only-precursors | 0.93 (0.72; 1.21) | 0.580 |
|  | All-class | 0.78 (0.54; 1.12) | 0.173 |
|  | All-neighbors | 0.94 (0.62; 1.42) | 0.767 |
| VLCSFA, very long chain saturated fatty acids; T2D, type-2 diabetes; HR, hazard ratio; CI, confidence interval.  The *basic* model was adjusted for age, waist circumference, height, leisure-time physical activity, smoking status, alcohol intake, education level, fasting status at blood draw, total energy intake, blood pressure (diastolic and systolic), standard clinical blood lipid markers (total cholesterol and triglycerides), antihypertensive medication, lipid-lowering medication, acetylsalicylic acid medication, and the respective sum of the lipid class. The *only-precursors* model was additionally adjusted for neighboring precursor lipids (shorter carbon chain length) within the same lipid class. The *all-class* model was further adjusted for all lipid neighbors within the same class. The *all-neighbors* model was adjusted for all direct neighbors. | | | |

**Supplemental Figures**


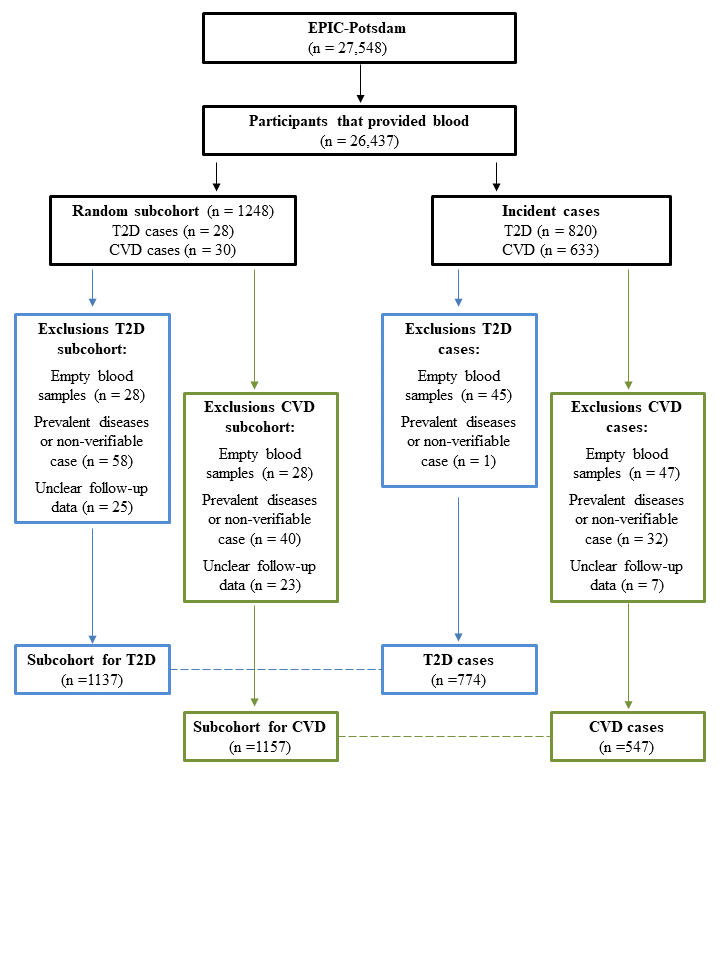


**Figure S1. Flow-chart of the T2D and CVD case-cohort design.**

T2D indicates type-2 diabetes and CVD cardiovascular disease.


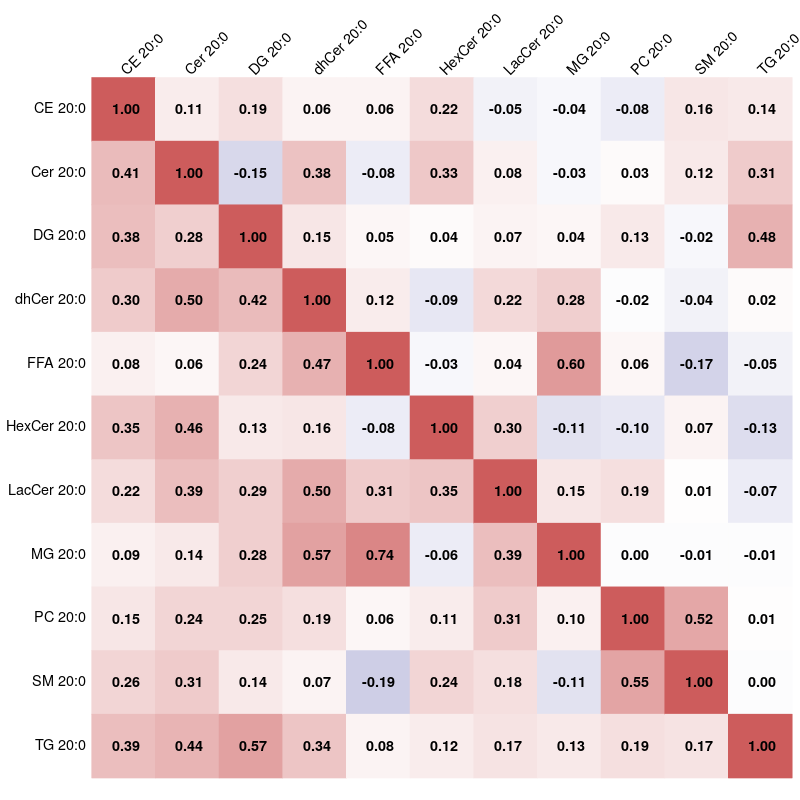


**Figure S2. Spearman rank correlation coefficients for C20:0 across lipid species.**

Lower-left triangle shows raw Spearman rank correlation coefficients, and upper-right triangle partial (adjusted for all other C20:0 molecular species) coefficients.

CE indicates cholesteryl esters; Cer, ceramides; DG, diacylglycerols; dhCer, dihydroceramides; FFA, free fatty acids; HexCer, hexosylceramides; LacCer, lactosylceramides; MG, monoacylglycerols; PC, phosphatidylcholines; SM, sphingomyelins; TG, and triacylglycerides.


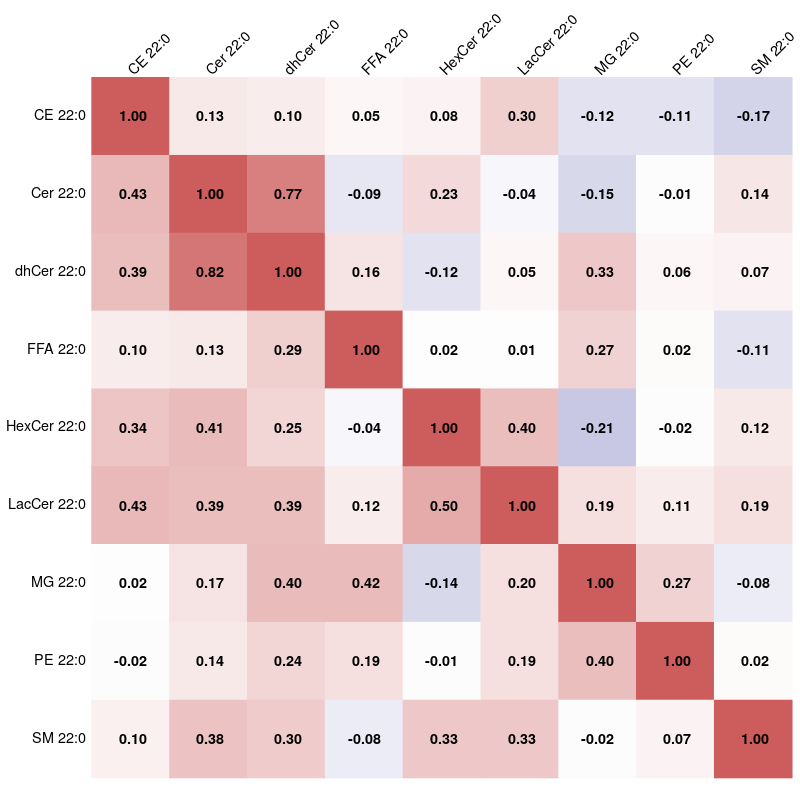


**Figure S3. Spearman rank correlation coefficients for C22:0 across lipid species.**

Lower-left triangle shows raw Spearman rank correlation coefficients, and upper-right triangle partial (adjusted for all other C22:0 molecular species) coefficients.

CE indicates cholesteryl esters; Cer, ceramides; dhCer, dihydroceramides; FFA, free fatty acids; HexCer, hexosylceramides; LacCer, lactosylceramides; MG, monoacylglycerols; PE, phosphatidylethanolamines; and SM, sphingomyelins.


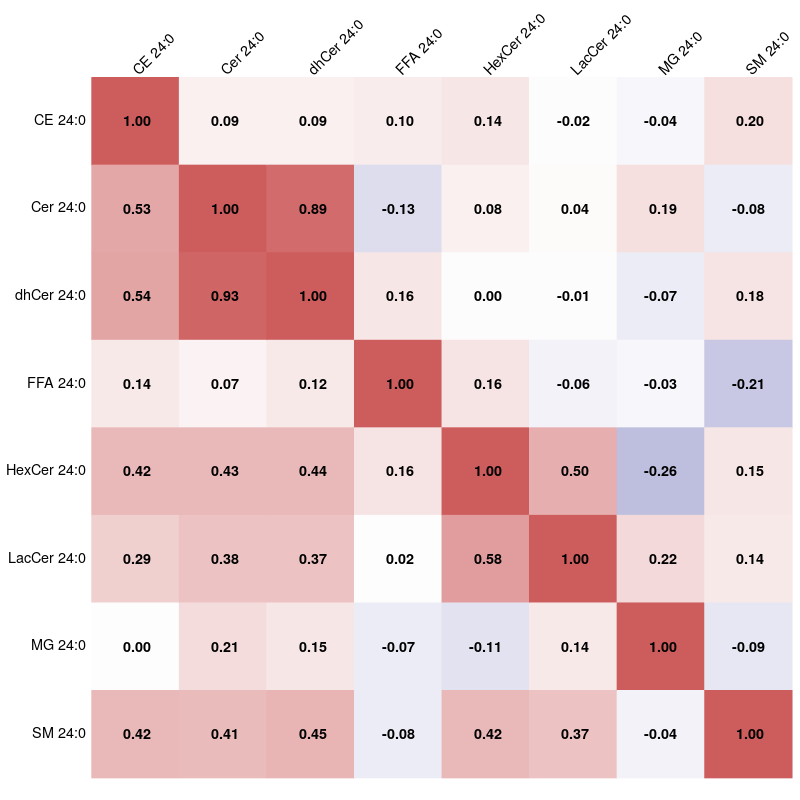


**Figure S4. Spearman rank correlation coefficients for C24:0 across lipid species.**

Lower-left triangle shows raw Spearman rank correlation coefficients, and upper-right triangle partial (adjusted for all other C24:0 molecular species) coefficients.

CE indicates cholesteryl esters; Cer, ceramides; dhCer, dihydroceramides; FFA, free fatty acids; HexCer, hexosylceramides; LacCer, lactosylceramides; MG, monoacylglycerols; and SM, sphingomyelins.


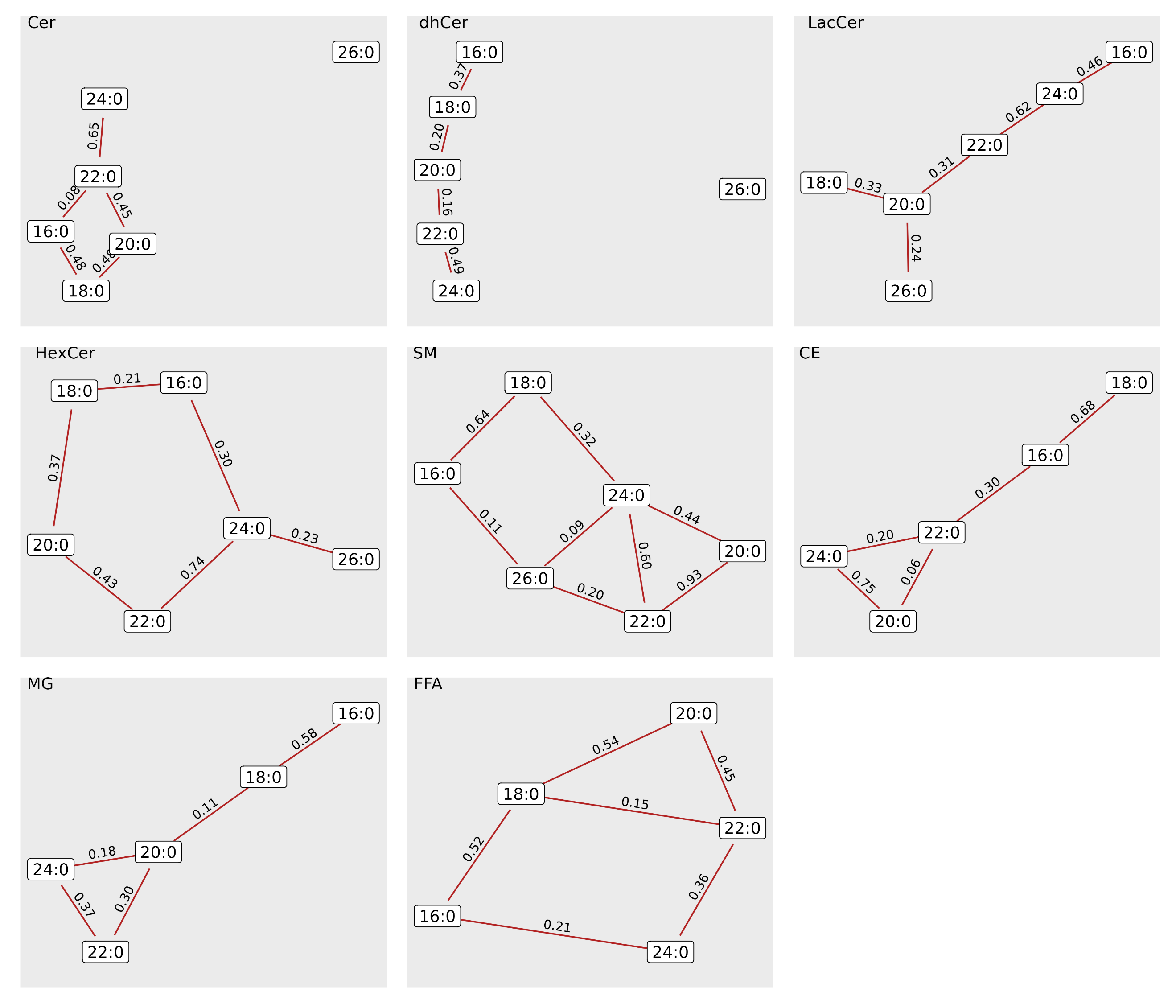
**Figure S5.** **Within class correlations (lipids from the same class with different saturated fatty acid).**

CE indicates cholesteryl esters; Cer, ceramides; dhCer, dihydroceramides; FFA, free fatty acids; HexCer, hexosylceramides; LacCer, lactosylceramides; MG, monoacylglycerols; and SM, sphingomyelins.
